# Priority Conceded

**Published:** 1914-01

**Authors:** C. W. Hennington

**Affiliations:** Rochester, N. Y.


### CORRESPONDENCE.

*This department is intended for the presentation of news and views not coming within the scope of other departments, and particularly to afford opportunity for discussion and criticism of views elsewhere expressed.*

### Priority Conceded.

Rochester, N. Y.

Editor Buffalo Medical Journal:

Please add a foot note to my paper on the modification of the Crile Canula, stating that I have just found a reference to the

*Annals of Surgery, October,* 1909, in which Bernstein describes a similar modification and should have the credit of priority.

Very truly yours,

C. W. HENNINGTON.

NOTE: The above was received just too late for insertion in the December issue.

